# Crystallography based structure elucidation of the complex of C-terminal fragment of Aβ polypeptide of Alzheimer’s disease with phospholipase A_2_

**DOI:** 10.1186/1471-2164-15-S2-P10

**Published:** 2014-04-02

**Authors:** Zeenat Mirza, Vikram Gopalakrishna Pillai, Sujata Sharma, Punit Kaur, Alagiri Srinivasan, Tej P Singh

**Affiliations:** 1 King Fahd Medical Research Center, P.O.Box 80216, King Abdulaziz University, Jeddah 21589, Kingdom of Saudi Arabia; 2 Department of Biophysics, All India Institute of Medical Sciences, New Delhi-110029, India; 3 Long Zheng Lab, Division of Pathology, 803I Abramson Research Center, The Children’s Hospital of Philadelphia, 34th Street and Civic Center Boulevard, Philadelphia, PA 19104, USA

## Background

Aggregation of Aβ polypeptide plays an important role in pathogenesis of Alzheimer’s disease, the most common form of dementia [1]. Therapies based on rationally designed aggregation inhibitors require knowledge of molecular structure [2]; because of high aggregation tendency of Aβ, it has not been possible to obtain complete structural information by X-ray crystallography. The hydrophobic C-terminal part of the Aβ peptide is critical in triggering transformation from α-helical to β-sheet structure. Phospholipases are an important enzyme involved in the inflammatory cascade mechanism having a conserved globular structure with active site, calcium binding loop, and hydrophobic channel. It has been speculated that phospholipase A_2_ (PLA_2_) inhibits the aggregation of Aβ peptide by interacting with the peptide and keeping the two peptide chains apart.

## Material and methods

PLA_2_ was purified to homogeneity from cobra venom. In order to examine the nature of interactions between PLA_2_ and Aβ_36-42_ peptide 1:1 complex of PLA_2_ with the C-terminal heptapeptide Val-Gly-Gly-Val-Val-Ile-Ala was prepared and co-crystallized. It is in tetragonal space group P4_1_ with unit cell dimensions, a=b=42.6 Å, c=65.8Å. X-ray intensity data were collected to 2.04 Å resolution. Structure has been determined by molecular replacement and refined to the crystallographic R factor of 0.193. Structural co-ordinates were deposited at RCSB’s PDB (3GCI).

## Results

Peptide binds to PLA_2_ at the hydrophobic substrate binding site and forms at least eight hydrogen bonds and about a two dozen Van der Waals interactions indicating that the affinity between PLA_2_ and the heptapeptide is far greater than the affinity between two Aβ peptide chains. Therefore, PLA_2_ may have a potential role to prevent the aggregation of Aβ peptides. Calcium has been found in the calcium binding site and has pentagonal bipyramidal geometry. Kinetic studies showed that the peptide VGGVVIA binds to PLA_2_ in a competitive manner with a binding constant of 5.2 x 10^-7^ M.

## Conclusions

This is the first attempt to structurally establish the interaction between Aβ peptide and PLA_2_. Results indicate that the peptide mainly adopts β-sheet secondary structure. Understanding the mechanism and effects of PLA_2_ upregulation in AD brain may help in the development of novel strategies to inhibit the inflammatory responses and delay AD progression. Preventing the folding of nascent Aβ monomer into toxic conformers or oligomers would have therapeutic benefits.
